# Clinical Indicators for Bacterial Co-Infection in Ghanaian Children with *P*. *falciparum* Infection

**DOI:** 10.1371/journal.pone.0122139

**Published:** 2015-04-09

**Authors:** Maja Verena Nielsen, Solomon Amemasor, Alex Agyekum, Wibke Loag, Florian Marks, Nimako Sarpong, Denise Dekker, Yaw Adu-Sarkodie, Jürgen May

**Affiliations:** 1 Bernhard Nocht Institute for Tropical Medicine, Infectious Disease Epidemiology, Hamburg, Germany; 2 Kumasi Centre for Collaborative Research in Tropical Medicine, Kumasi, Ghana; 3 Kwame Nkrumah University of Science and Technology, School of Medical Sciences, Kumasi, Ghana; 4 International Vaccine Institute, Seoul, South Korea; 5 German Center for Infection Research (DZIF), partner site Hamburg-Borstel-Lübeck, Hamburg, Germany; Institut de Recherche pour le Développement, FRANCE

## Abstract

Differentiation of infectious causes in severely ill children is essential but challenging in sub- Saharan Africa. The aim of the study was to determine clinical indicators that are able to identify bacterial co-infections in *P*. *falciparum* infected children in rural Ghana. In total, 1,915 severely ill children below the age of 15 years were recruited at Agogo Presbyterian Hospital in Ghana between May 2007 and February 2011. In 771 (40%) of the children malaria parasites were detected. This group was analyzed for indicators of bacterial co-infections using bivariate and multivariate regression analyses with 24 socio-economic variables, 16 terms describing medical history and anthropometrical information and 68 variables describing clinical symptoms. The variables were tested for sensitivity, specificity, positive predictive value and negative predictive value. In 46 (6.0%) of the children with malaria infection, bacterial co-infection was detected. The most frequent pathogens were non-typhoid salmonellae (45.7%), followed by *Streptococcus* spp. (13.0%). Coughing, dehydration, splenomegaly, severe anemia and leukocytosis were positively associated with bacteremia. Domestic hygiene and exclusive breastfeeding is negatively associated with bacteremia. In cases of high parasitemia (>10,000/μl), a significant association with bacteremia was found for splenomegaly (OR 8.8; CI 1.6–48.9), dehydration (OR 18.2; CI 2.0–166.0) and coughing (OR 9.0; CI 0.7–118.6). In children with low parasitemia, associations with bacteremia were found for vomiting (OR 4.7; CI 1.4–15.8), severe anemia (OR 3.3; CI 1.0–11.1) and leukocytosis (OR 6.8 CI 1.9–24.2). Clinical signs of impaired microcirculation were negatively associated with bacteremia. Ceftriaxone achieved best coverage of isolated pathogens. The results demonstrate the limitation of clinical symptoms to determine bacterial co-infections in *P*. *falciparum* infected children. Best clinical indicators are dependent on the parasitemia level. Even with a moderate sensitivity of >60%, only low positive predictive values can be obtained due to low prevalence of bacteremia. Rapid testing for distinguishing parasitemia and bacteremia is essential.

## Background

Differentiation of infectious causes of febrile illnesses in children presenting to hospitals in rural sub-Saharan areas is a challenge for clinicians, in particular in co-infections [1,2]. Due to an immature immune system, especially children tend to react with increasing body temperatures to various infectious agents. The synonymously use of *fever* and *malaria* is common in the general public and also plays an important role as presumptive diagnosis of clinicians.

Reasons for misdiagnosis can partly be accredited to overlapping clinical symptoms of malaria and acute bacterial infections as well as lacking diagnostic tools [3]. Deficient education of laboratory staff complicates the diagnostic process [4].

Recent studies evaluating WHO guidelines for antimicrobial treatment in children admitted to the hospital in an area of intense *Plasmodium falciparum* transmission reveals that guidelines failed to identify almost a third of bacteremic children [1].

According to WHO publication of 2011, Ghana is considered to be a high transmission area (≥ 1 case per 1,000 inhabitants) for Malaria, which might contribute to demonstrable frequent misinterpretation of acute systemic bacterial infection for malaria resulting in inadequate treatment of bacteremia [5]. Recent data of settings with microscopy-based malaria diagnostics suggests that interpretation of parasite slides often generates false positive results, accounting for over-diagnosis of malaria [6]. Especially in the case of co-infection, bacteremia tends to be missed after detection of parasites.

However, previous investigation of the study site revealed a surprisingly high percentage of almost 20% bacteremic children below the age of five years who were hospitalized due to severe non-traumatic illness [7]. These results underline the need of a focused investigation of bacteremia in high transmission areas of malaria and respective patterns of clinical occurrence in case of co-infection.

We report the results of a hospital-based study on severe illness of children, describing a population exposed to intense *P*. *falciparum* transmission. Our aim is to present clinical signs and symptoms of children, which could help to clinically identify bacterial co-infections in malaria slide positive children below 15 years of age in rural Ghana and which could complement information to current guidelines.

## Methods

Children were recruited at the Child Welfare Clinic of the Agogo Presbyterian Hospital (APH), a local teaching hospital in the Ashanti Region, Ghana, between May 2007 and February 2011 (46 months). The hospital catchment area encompasses approximately 149,500 people [8], with more than 28,000 inhabitants living in the town Agogo [9]. The area is holoendemic for malaria with reported transmission rates of >100 per 1,000 inhabitants in 2008 [10]. Predominating species is *P*. *falciparum*, contributing to 92.4% of malarial infections [11] with two seasonal peaks [12].

In the study area, bacterial blood stream infections are responsible for one-fifth of hospital admissions in severely ill children below 5 years of age. Non-typhoidal salmonellae (NTS) contribute to more than half of infections (53.3%), leading to a cumulative incidence of 25.2 (CI 21.1–29.4) per 1,000 per year [7]. A National Health Insurance Scheme (NHIS) was officially launched in March 2004, covering 38% of the district population in 2009 [13]. The HIV prevalence was 1.8% for the adult Ghanaian population in 2009 [14]. In 2001, *Haemophilus influenzae* type B conjugate vaccine was introduced in the immunization schedule for children [15]. At the time of investigation, no routine pneumococcal vaccination was available, which was introduced in the national immunization program of Ghana in April 2012.

First-line treatment for severe malaria was parenteral quinine followed by oral administration. Antibiotics were not routinely used in treatment of all children admitted for malaria unless sepsis was also suspected on the basis of clinical parameters, in which case ceftriaxone, gentamicin plus ampicillin for pneumonia, or co-amoxiclav/cloxacillin plus metronidazole for sepsis resulting from abscess, or other combinations were used as first line treatments, with ceftriaxone as the main first choice in doubtful cases. Modifications or additions of the antibiotic regimen were made based on the clinical scenarios and results of bacteriologic culture and sensitivity testing.

### Recruitment

All patients below the age of 15 years who required admission because of severe illness were recruited. Exclusion criteria were defined as obvious non-infectious cause for admission, such as trauma, elective surgery, congenital abnormalities and exclusive dermatological cases.

Aims and principles of the study were explained in detail to participants and informed consent was given by signature or thumb print by the caregiver. Ethical approval for the study was obtained from the Committee on Human Research, Publications, and Ethics, School of Medical Science, Kwame Nkrumah University of Science and Technology (KNUST), Kumasi, Ghana.

### Clinical and laboratory data collection

Data collection was entirely embedded into the clinical routine. On admission of children, history and clinical data was recorded on standardized charts containing a 4-paged admission sheet, including a registration form and a 3-paged clinical recruitment chart. The set of clinical information contained WHO diagnostic criteria for septicemia in children and sepsis criteria of the international pediatric sepsis conference [16,17].

Socio-economic data such as educational background, financial situation and everyday life (e.g. housing, cooking, religion, health precautions) of the patient’s family was assessed by interviewing a parent or the guardian with a structured questionnaire in English or the local language, Twi.

Standardized malaria parasite diagnosis was performed for each patient on admission, including thick and thin smears from capillary blood samples, described elsewhere (2).

1ml blood for aerobic and anaerobic blood cultures was taken at the children’s ward through phlebotomy after alcoholic skin disinfection. The blood was inoculated into Becton Dickinson (BD) BACTEC PEDS PLUS/F bottles containing 20 ml of enriched broth with resin using a separate needle to pierce the rubber lid of the bottle after it had been cleaned with 70% Ethanol and allowed to dry. Bottles were incubated in a BD BACTEC 9050 Blood Culture system (BD Diagnostics, Sparks, Massachusetts, USA) [8]. Every positive vial flagged by the system was subcultured on standard media for species differentiation. Broth from the positive bottle was examined by direct methods, such as Gram stain. Isolates were identified based on colonial morphology, biochemical tests, Analytical Profile Index (API) reaction and serology as necessary. The sensitivity of amoxicillin/ampicillin, amoxiclav (amoxicillin & clavulanic acid), cefuroxime, ceftriaxone, co-trimoxazole, ciprofloxacin, gentamicin, tetracycline and chloramphenicol was tested using the Kirby-Bauer disc diffusion method following CLSI guidelines. *S*. *enterica* isolates were screened for resistance to fluoroquinolones by nalidixic acid disc diffusion following the Clinical and Laboratory Standards Institute (CLSI) guidelines of 2011. Nalidixic acid resistant strains were further tested by ciprofloxacin E test. When referring to *Streptococcus* spp., it does not include *S*. *pneumoniae* throughout this study. Measurement of hemoglobin was conducted semi-automatic via Sysmex-tool.

### Data entry and data cleaning

Admission charts were filled in by doctors or study nurses and subsequently double entered by two independent data entry clerks using a 4^th^ Dimension Database 2004.4 4D SA, 1985–2006. (Clichy-la-Garenne, France).

Initially, 512 crude variables covering socio-economic background, medical history and acute clinical symptoms were derived from questionnaires. Patients with missing data for sex, age, bacteriological results or malaria diagnostics were excluded.

Continuous variables (e.g. “*heart rate”*) were categorized in generally accepted clinical subgroups (e.g. “*bradycardia”/“normocardia”/“tachycardia”*). In some cases, variables were merged in order to establish a new, medically more reasonable variable, or to improve statistical power of variables with small case numbers (e.g. “*bulging fontanel”* and/or “*stiff neck*” combined to “*clinical signs of meningitis”*). Redundant, similar or variables with >50% missing values were excluded.

In order to create a variable that can be used to describe the socio-economic status of the patient’s households, we applied a principal component analysis (PCA) on the following variables: mother’s and father’s profession and education, type of homestead, water supply, existence of an indoor kitchen, electricity, indoor toilet, use of freezing as measure of conservation, existence of a relative abroad who might financially support the family, the self-rated ability to manage with the available monthly income as well as existing health insurance. The factor that showed the highest eigenvalue was divided in three tertiles, which accordingly represent three categories of socio-economic status, which we labeled “*poor/average/high”*. For further details see [18]. Single socio-economic values were still considered for further analysis.

After data cleaning, 24 socio-economic variables, 16 terms describing medical history and anthropometrical variables and 68 variables describing clinical signs and symptoms were effectively used for further analysis (see S1 Table).

### Statistical analysis

To screen variables for their influence on bacteremia in parasitemic children, we applied three consecutive analytical steps with bacteremia as the dependent variable using STATA 10 software (College Station, Texas, United States). First, bivariate regressions analysis was applied to all socio-economic, medical history and anthropometrical variables, as well as to all clinical signs and symptoms (see S1 Table). Basic data and major variables describing the socio-economic background of the children’s families are displayed in Table 1. Variables of p-value ≤ 0.1 were than identified for further analysis in a multivariate regression model. This selective step was necessary, since multivariate regression analysis of all variables was not possible due to a large volume of variables attended by a small common denominator due to heterogeneous patterns of missing data. Unstratified results of bivariate and multivariate regression analysis (variables with identified p-value ≤ 0.1 in bivariate model) of all children with co-infection are shown in Table 2. Variables with a sample size <300 cases were excluded from analysis for a better significance of the model. The multivariate model was applied to eliminate confounders and correlation across variables.

**Table 1:** Basic data of the study group and socio-economic status.

Characteristic		Total (%)	Malarial parasite +ve (%)	Malarial parasite -ve (%)
Sex (n = 1,915)	Female	866 (45.2)	355 (46.0)	511(44.7)
	Male	1,049 (54.8)	416 (54.0)	633 (55.3)
Age (n = 1,915)	0–1 month	127 (6.6)	8 (1.0)	119 (10.4)
	1–11 months	429 (22.4)	108 (14.0)	321 (28.0)
	1-<5 years	947 (49.5)	501 (65.0)	446 (39.0)
	5-<15 years	412 (21.5)	154 (20.0)	258 (22.6)
	Median (IQR)	24 (11–54)	32	20
Religion (n = 1,607)	Christian	1,338 (83.3)	559 (82.5)	779 (83.8)
	Moslem	197 (12.2)	85 (12.5)	112 (12.1)
	Other	72 (4.5)	34 (5.0)	38 (4.1)
Ethnicity (n = 1,911)	Akan	1,344 (70.3)	521 (67.6)	823 (72.1)
	Northerners	444 (23.2)	203 (26.4)	241 (21.1)
	Others	123 (6.5)	46 (6.0)	77 (6.8)
Access to electricity (n = 1,607)	No	652 (40.6)	275 (40.6)	377 (40.6)
	Yes	955 (59.4)	403 (59.4)	552 (59.4)
Water supply (n = 1,606)	River/well	283 (17.6)	113 (16.7)	170 (18.3)
	Tap/pipe	1.323 (82.4)	565 (83.3)	758 (81.7)
House type (n = 1,607)	Wood/Mud	423 (26.3)	179 (26.4)	244 (26.3)
	Cement/brick	1,184 (73.7)	499 (73.6)	685 (73.7)
Mother literate (n = 776)	No	568 (73.2)	199 (80.6)	369 (69.8)
	Yes	208 (26.8)	48 (19.4)	160 (30.2)
Father literate (n = 773)	No	265 (34.3)	94 (38.2)	171 (32.4)
	Yes	508 (65.7)	152 (61.8)	356 (67.6)
Health insurance (n = 1,604)	No	523 (32.6)	249 (36.7)	274 (29.6)
	Yes	1,081 (67.4)	429 (63.3)	652 (70.4)
Income management (n = 1,605)	„Not difficult“	414 (25.8)	139 (20.6)	275 (29.6)
	„Difficult“	1,191 (74.2)	537 (79.4)	654 (70.4)

**Table 2:** Unstratified bivariate and multivariate analysis of characteristics associated with bacteremia in parasitemic children <15 years (with p < 0.1).

Characteristics				Bacteremia			
				Bivariate analysis		Multivariate analysis^5^ (n = 377)	
		No	Yes (%)	OR (CI)^1^	p-value	OR (CI)^1^	p-value
Age (n = 771)	<5 years	575	42 (6.8)	1		*	
	5 - <15 years	150	4 (2.6)	0.4 (0.1–1.0)	0.06		
Developmental delay^2^ (n = 145)	No	129	2 (1.5)	1		-	
	Yes	12	2 (14.3)	10.8 (1.4–83.3)	0.02		
Exclusive breastfeeding (n = 187)	No	111	14 (11.2)	1		-	
	Yes	61	1 (1.6)	0.1 (0.0–1.0)	0.05		
History of cough (n = 767)	No	526	25 (4.5)	1		1	
	Yes	196	20 (9.3)	2.1 (1.2–4.0)	0.01	19.3 (0.0–15428.3)	0.39
Able to breastfeed (n = 656)	No	403	28 (6.5)	1		1	
	Yes	219	6 (2.7)	0.4 (0.2–1.0)	0.04	1.9 (0.4–9.1)	0.40
Convulsions (n = 749)	No	476	39 (7.6)	1		1	
	Yes	224	7 (3.0)	0.4 (0.2–0.9)	0.02	0.5 (0.1–2.7)	0.42
Circulation impaired^3^ (n = 722)	No	42	7 (14.3)	1		*	
	Yes	644	29 (4.3)	0.3 (0.1–0.7	<0.001		
Capillary refill time ≥2 (n = 715)	No	144	23 (13.8)	1		1	
	Yes	536	12 (2.2)	0.1 (0.1–0.3)	<0.001	2.6 (**)	**
Cough (n = 767)	No	477	23 (4.6)	1		1	
	Yes	245	22 (8.2)	1.9 (1.0–3.4)	0.04	0.1 (0.0–68.4)	0.47
Watery stool (n = 295)	No	143	22 (13.3)	1		-	
	Yes	121	9 (6.9)	0.5 (0.2–1.1)	0.08		
Thirsty drinking (n = 766)	No	303	30 (9.0)	1		1	
	Yes	418	15 (3.5)	0.4 (0.2–0.7)	<0.001	1.0 (0.2–5.0)	0.98
Splenomegaly (n = 573)	No	497	11 (2.2)	1		1	
	Yes	58	7 (10.8)	5.5 (2.0–14.6)	<0.001	1.3 (1.3–1.4)	<0.001
Dehydration^4^ (n = 703)	No	50	2 (3.9)	1		1	
	Yes	656	32 (4.7)	5.1 (1.4–19.1)	0.02	206.0 (4.5–9503.9)	0.006
Leukocytosis >10,000/μl (n = 768)	No	456	18 (3.8)	1		1	
	Yes	266	28 (9.5)	2.7 (1.4–4.9)	<0.001	0.9 (0.2–4.5)	0.94
Severe anemia <8 mg/dl (n = 769)	No	442	18 (3.9)	1		1	
	Yes	281	28 (9.1)	2.5 (1.3–4.5)	<0.001	2.3 (0.5–10.8)	0.28
Parasitemia (n = 626)	<10,000/μl	153	15 (8.9)	1		1	
	>10,000/μl	441	17 (3.7)	0.4 (0.2–0.8)	0.01	0.2 (0.1–1.1)	0.06
Mosquito net (n = 676)	No	523	33 (5.9)	1		1	
	Bed/ window net	119	1 (0.1)	0.1 (0.0–1.0)	0.05	0.5 (0.0–5.4)	0.54
Washing their hands (n = 677)	Not before meals	50	8 (13.8)	1		1	
	Before meals	592	27 (4.4)	0.3 (0.1–0.7)	<0.001	0.25 (0.0–1.6)	0.14

^1^ OR (CI), odds ratio (95% confidence interval)

^2^ Children who cannot hold their head at the age of three months, roll over at the age of 6 months, sit unsupported at the age of 9 months, stand unsupported at the age of 12 months or walk single steps at the age of 18 months

^3^ Circulation impaired = cold extremities and/or capillary refill time >2 sec. and/or tachycardia

^4^ Dehydration ≥dehydration grade 1 (3–5%)

^5^ Variables *developmental delay*, *exclusive breastfeeding* and *watery stool* were removed from multivariate analysis due to small case number (<300 cases)

* Predicts failure perfectly

** No calculation possible due to small case numbers

Second, we performed bivariate regression analysis for the respective subgroups of children stratified for parasite count (*<10*,*000 parasites per μl blood/≥10*,*000 parasites per μl blood*) (variables of p-value ≤ 0.1 in bivariate model shown in Table 3). Out of these two sets of variables, all factors positively associated with co-infection (“positive clinical predictors”) were then entered into a parsimonious stepwise multivariate regressions model to identify the strongest factors associated with co-infection (Table 4).

**Table 3:** Bivariate analysis of characteristics associated with bacteremia in parasitemic children <15 years stratified for parasite count (with p < 0.1).

Characteristics		Bacteremia							
		<10,000 parasites/μl				>10,000 parasites/μl			
		No	Yes (%)	OR (CI)	p-value	No	Yes (%)	OR (CI)	p-value
Intake of antimalarials	No	135	10 (6.9)	1		396	14 (3.4)	1	
	Yes	17	5 (22.7)	4.00 (1.21–13.18)	0.02	38	1 (2.6)	0.74 (0.09–5.76)	0.77
History of cough	No	107	11 (9.3)	1		324	7 (2.1)	1	
	Yes	46	4 (8)	0.86 (0.26–2.86)	0.81	115	9 (7.3)	3.58 (1.31–9.84)	0.01
Cold extremities	No	-	-	1		421	14 (3.2)	1	
	Yes	-	-	-	-	4	1 (20.0)	7.66 (0.80–73.05)	0.077
Capillary refill time ≥2	No	21	8 (27.6)	1		70	10 (12.5)	1	
	Yes	128	5 (3.8)	0.11 (0.03–0.36)	0.000	349	4 (1.1)	0.08 (0.02–0.26)	0.000
Abnormal skin	No	-	-	1		165	9 (5.2)	1	
	Yes	-	-	-	-	7	3 (30.0)	7.90 (1.75–35.77)	0.01
High fever ≥ 38.5°C	No	90	6 (6.3)	1		177	11 (5.9)	1	
	Yes	63	9 (12.5)	2.19 (0.74–6.47)	0.16	264	6 (2.2)	0.37 (0.13–1.01)	0.05
Circulation impaired^1^	No	7	3 (30.0)	1		20	4 (16.7)	1	
	Yes	142	10 (6.6)	0.17 (0.04–0.77)	0.02	404	11 (2.7)	0.13 (0.04–0.46)	0.001
Cough	No	99	11 (10.0)	1		290	6 (2.0)	1	
	Yes	54	4 (6.9)	0.69 (0.21–2.28)	0.54	150	10 (6.3)	3.18 (1.14–8.92)	0.03
Drawing-in of chest wall	No	-	-	1		430	15 (3.4)	1	
	Yes	-	-	-	-	9	2 (18.2)	5.83 (1.17–28.95)	0.03
Respiratory distress	No	-	-	1		409	13 (3.1)	1	
	Yes	-	-	-	-	13	2 (13.3)	4.93 (1.01–24.15)	0.05
Heavy diarrhoea^2^	No	14	1 (6.7)	1		54	2 (3.6)	1	
	Yes	4	3 (42.9)	14.00 (1.06–185.49)	0.05	28	1 (3.5)	0.93 (0.08–10.71)	0.95
Vomiting	No	102	5 (4.7)	1		222	8 (3.5)	1	
	Yes	51	10 (16.4)	1.02 (0.38–2.76)	0.97	218	8 (3.5)	4.17 (1.35–12.89)	0.01
Vomiting everything	No	32	3 (8.6)	1		125	6 (4.6)	1	
	Yes	19	6 (24.0)	3.53 (0.78–15.95)	0.10	91	2 (2.2)	0.46 (0.09–2.31)	0.34
Tenting of skin	No	-	-	1		423	14 (3.2)	1	
	Yes	-	-	-	-	2	1 (33.3)	15.39 (1.32–179.95)	0.03
Splenomegaly	No	121	5 (4.0)	1		331	3 (0.9)	1	
	Yes	12	1 (7.7)	1.88 (0.20–17.48)	0.58	27	4 (12.9)	16.74 (3.56–78.67)	0.00
Dehydration^3^	No	-	-	1		411	12 (2.8)	1	
	Yes	-	-	-	-	6	3 (33.3)	17.46 (3.90–78.24)	0.00
White blood cell count	Normocytic	83	3 (3.5)	1		227	5 (2.2)	1	
	Leukocytosis^4^	50	11 (18.0)	6.16 (1.64–23.23)	0.01	160	12 (7.0)	3.40 (1.18–9.84)	0.02
	Leukopenia	17	1 (5.6)	1.65 (0.16–16.85)	0.68	54	0 (0.0)	-	-
Severe anemia <8 mg/dl	No	95	5 (5.0)	1		281	9 (3.1)	1	
	Yes	56	10 (15.2)	3.61 (1.17–11.13)	0.03	160	8 (4.8)	1.53 (0.58–4.04)	0.39
Toilet in house	No	75	9 (10.7)	1		184	9 (4.7)	1	
	Yes	63	4 (6.0)	0.52 (0.15–1.77)	0.29	218	3 (1.4)	0.29 (0.08–1.07)	0.06
Socioeconomic status	Low	46	8 (14.8)	1		134	4 (2.9)		
	Medium	49	2 (3.9)	0.23 (0.05–1.14)	0.07	124	5 (3.9)	1.36 (0.36–5.17)	0.65
	High	51	4 (7.3)	0.42 (0.12–1.49)	0.18	166	6 (3.5)	1.24 (0.34–4.48)	0.74

^1^ Circulation impaired = cold extremities and/or capillary refill time ≤2 sec. and/or tachycardia

^2^ Diarrhea >7 days

^3^ Dehydration ≥dehydration grade 1 (3–5%)

^4^ Leukocytosis = white blood cell count ≥10,000/μl; Leukopenia = white blood cell count <4,000/μl

**Table 4:** Parsimonious multivariate regressions model of characteristics positively associated with bacteremia in parasitemic children, stratified for parasite count.

		Risk for bacteremia	
		OR (CI)	^1^p-value
**Parasitemic children with <10,000 parasites/**μ**l (n = 164)**			
Severe anemia <8 mg/dl	No	1	
	Yes	3.3 (1.0–11.1)	0.05
Vomiting	No	1	
	Yes	4.7 (1.4–15.8)	0.01
Leukocytosis >10,000/μl	No	1	
	Yes	6.8 (1.9–24.2)	0.00
**Highly parasitemic children with ≥10,000 parasites/**μ**l (n = 357)**
Cough	No	1	
	Yes	9.0 (0.7–118.6)	0.10
Dehydration^2^	No	1	
	Yes	18.2 (2.0–166.0)	0.01
Splenomegaly	No	1	
	Yes	8.8 (1.6–48.9)	0.01

^1^ OR (CI), odds ratio (95% confidence interval)

^2^ Dehydration ≥ grade 1 (3–5%)

Third, positive and negative predictive values of resulting factors were calculated, displayed in Table 5. Negative and positive predictive values were compiled for each of six variables using STATA 10 software.

**Table 5:** Test accuracy.

	Prevalence %	Sensitivity % (CI)	Specificity % (CI)	PPV (%)^1^	NPV (%)^2^
**Parasitemic children with <10,000 parasites/**μ**l**					
Severe anemia <8 mg/dl (n = 166)	9.0	67 (38–88)	63 (55–71)	15	95
Vomiting (n = 168)	8.9	67 (38–88)	67 (59–74)	16	95
Leukocytosis >10,000/μl (n = 165)	9.1	73 (45–92)	67 (59–74)	18	96
**Highly parasitemic children with ≥10,000 parasites/**μ**l**					
Cough (n = 456)	3.5	63 (35–85)	66 (61–70)	6	98
Dehydration^3^ (n = 432)	3.5	20 (4–48)	99 (97–100)	33	97
Splenomegaly (n = 365)	1.9	57 (18–90)	93 (89–95)	13	99

^1^ PPV, positive predictive value for bacteremia (for prevalence 12.7%)

^2^ NPV, negative predictive value for bacteremia (for prevalence 12.7%)

^3^ Dehydration ≥ grade 1 (3–5%)

## Results

**Fig 1:**
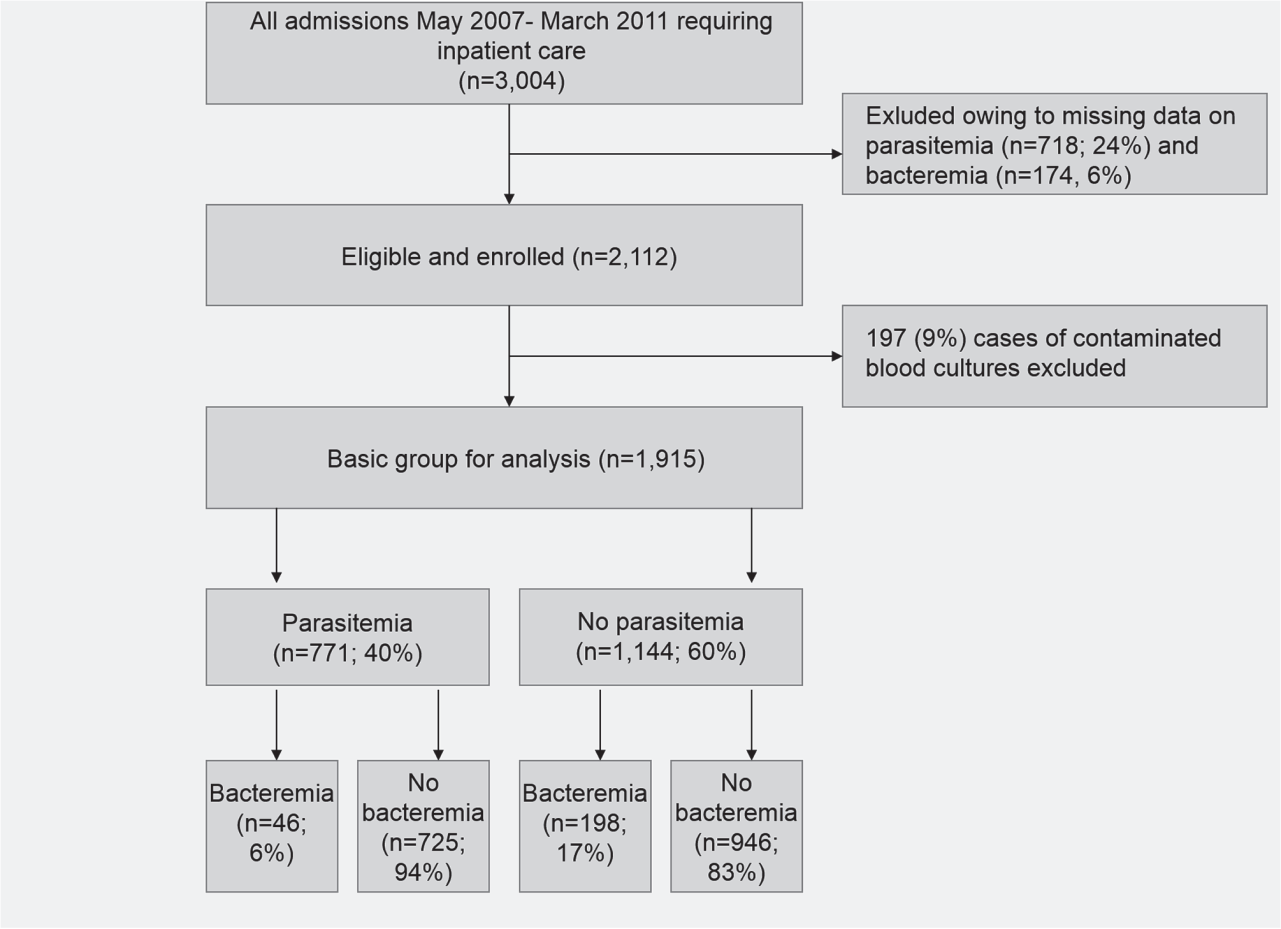
Admissions, enrollment, frequencies and percentages of parasitemia/bacteremia.

In total, 13,399 children below the age of 15 years were admitted to the children outpatient department of Agogo Presbyterian Hospital between May 2007 and March 2011. Out of 3,004 (22.4%) children requiring inpatient care due to severe febrile or non-febrile internal illness, 718 cases (23.9%) were excluded because of missing malaria slides, of which another 174 (5.8%) were excluded since bacteriological results were missing. Of remaining 2,112 patients, 197 (9.3%) showed a pathogen spectrum indication contamination, leaving 1,915 for further analysis (Fig 1).

The proportion of female (45%) and male (55%) children in the study population was comparable. The age pattern showed a median of 24 and a mean of 39 months. Seventy-eight percent (n = 1,503) of children were younger than five years, 29% (n = 556) younger than 1 year and 7% (n = 127) younger than one month (Table 1).

Malaria parasitemia was detected in 771 (40.3%) children, whereas 458 (73.2%) out of all counted slides showed parasite concentrations >10,000 parasites/ml and 195 (31.3%) concentrations above the WHO defined threshold of 4% for hyperparasitemia.

In 46 (6.0%) of parasitemic cases, bacteremia was detected in blood samples indicating a co-infection. In 69.6% (n = 32) of co-infections, data on parasite count (amount of parasites per μl blood) was available. A low parasite count (<10,000/μl) was found in 46.9% (n = 15) and a high parasite count in 53.1% (n = 17) of cases.

Comparable to earlier published data of the same study site [7], NTS dominates the isolated pathogens accounting for 45.7% (n = 21) of all isolates in parasitemic children <15 years, followed by *Streptococcus* spp. (n = 6; 13.0%), *S*. *aureus* (n = 4; 8.7%), *S*. *pneumoniae* and *Pseudomonas* spp. (n = 3; 6.5%, respectively). *Salmonella ser*. Typhi, *Escherichia coli* and *Acinetobacter* spp. accounted for two isolates each (4.4% respectively). One isolate (2.2%) was detected for *Klebsiella* spp., *Shigella* spp. and *Chryseobacter* spp. each. In children below the age of five years, the frequency of the three most prevalent species remained equal.

Comparing the subgroups of under five children with parasite count below 10,000μl blood with those showing a high parasitemia, the dominating effect of NTS in those with a low parasitemia is more distinct (n = 7; 53.9%) than in the other group (n = 7; 43.8%). While *Streptococcus* spp. and *Staphylococcus aureus* played a minor role in children with low parasitemia (n = 1; 7.7%, respectively), they accounted for the three most frequent isolates in cases with high parasitemia (n = 4; 25.0% and n = 3; 18.8% respectively).

Invasive bacterial infection was more likely in slide negative children (n = 198; 17.3%) than in slide positive children (n = 46; 6.0%). Compared to the group of children with a negative parasite count (n = 198), *Streptococcus* spp. is represented more than ten times more frequently in the group of parasitemic children. On the other hand, *Salmonella ser*. Typhi only accounts for 4.4% (n = 2) in parasitemic children, whereas 17.7% (n = 35) of isolates were detected in non-parasitemic children. Similar effects were seen for *S*. *pneumoniae* that accounted for 12.6% in non-parasitemic (n = 25), but only for 6.5% (n = 3) in parasitemic children. NTS and *S*. *aureus* showed similar percentages in non-parasitemic children (40.4%; n = 80 and 11.6%; n = 23%) compared to the children with positive parasite count.

The outcome of disease at discharge was documented in 89.4% (n = 1,712) of cases. Other unknown underlying conditions of children might have influenced recovery and clinical expression of bacterial infection. Cases of co-infection were fatal in 1 child (2.2%), 2 children suffered from ongoing disabilities (4.3%) and 12 children (67.4%) fully recovered.

Overall, mortality of bacteremic children was 7.0% (n = 13) compared to 2.8% (n = 42) of children without invasive bacterial blood stream infection. Isolates from fatal cases were four NTS, three *S*. *pneumoniae*, *two S*. *aureus*, two *Klebsiella* spp., one *Salmonella* ser. Typhi and one *Acinetobacter* spp.

In the only fatal case of co-infection, the child was highly parasitemic. High parasitemia was found in 75% of non-bacteremic children who died during hospitalization.

NTS, *S*. *pneumoniae*, *Pseudomonas* spp. and *Salmonella* ser. Typhi were 100% susceptible to ceftriaxone, whereas ciprofloxacin fully covered NTS, *S*. *aureus* and *Pseudomonas* spp. Sixty-seven percent of *S*. *pneumoniae* were resistant towards ciprofloxacin. *Streptococcus spp*., the second frequent isolate showed 20% resistance to ceftriaxone and 33% resistance to ciprofloxacin. If presenting with symptoms of diarrhea and respiratory infection, ceftriaxone fully covered isolated pathogens. In 85% of NTS, 17% of *Streptococcus* spp. and 100% of *Pseudomonas* spp., amoxicillin was tested resistant. *S*. *pneumoniae* has not built resistance yet, but was partly intermediately susceptible. Gentamicin only covered 48% of NTS and 50% of *S*. *pneumoniae* isolates. Multi drug resistance against amoxicillin, chloramphenicol and cotrimoxazole was detected in one NTS und one *Salmonella* ser. Typhi strain.

When evaluating presumption diagnosis after clinical examination but without knowledge of laboratory testing results, malaria was suspected in 1,272 cases (66.4%), whereas bacteremia/sepsis was suspected in 1,155 cases (60.3%). Co-infection, respectively strong suspicion of both diagnoses, was documented in 852 cases (48.2%). During primary inspection, if health care professionals suspected an isolated malaria infection, 43.5% (n = 266) actually were parasitemic, whereas 7.5% (n = 46) showed positive blood cultures without clinical prediction. On the other hand, 7.4% (n = 37) out of suspected bacteremic cases were predicted correctly, and 6.0% (n = 30) of parasitemia could not be clinically detected.

### Clinical factors associated with bacteremia in parasitemic children

Applying bivariate regression analysis to the 107 derived anamnestic and clinical characteristics, 18 showed a significant association (p-value<0.1) with bacteremia in parasitemic children (Table 2).

Coughing or a history of coughing seems to increase the risk of suffering from bacteremia in this study group. Dehydration (dehydration >grade 1≈3–5%) as well as splenomegaly as an unspecific reaction to acute or chronic infectious condition show a strong positive association with bacteremia. As laboratory parameters, severe anemia (<8 mg/dl) and leukocytosis (>10,000/μl) indicate a strong association with bacteremia in parasitemic children. This effect dissolves for severe anemia when stratifying for cases with parasitemia of >10,000/μl only (Table 3). A general developmental delay of the sick child can also be evidence of an additional bacteremia. In multivariate analysis of significant factors in bivariate analysis, splenomegaly and dehydration remain significantly associated with a co-infection, whereas all other factors lose significance (Table 2).

In our analysis, the calculated variable of “socio-economic status” showed a slight negative association of medium and high social standard with bacterial co-infection in bivariate model but this association turned out to be irrelevant upon further analysis. Other particular socio-economic aspects, like the domestic application of procedures improving hygiene (e.g. washing their hands before eating), are negatively associated with bacteremia in this age group. Furthermore, the utilization of mosquito nets has a strong protective influence on bacteremia. The risk of bacteremia is decreased if children have exclusively been breastfed till onset of the disease or at least were able to breastfeed with or without intake of solid food or were simply strong enough to drink thirstily. Anamnestic private preadmission intake of antimalarials without reduction of signs of severe illness in children with low parasite count is an indicator for clinicians of invasive bacterial disease.

Interestingly, signs of impaired microcirculation, like prolonged capillary refill time or cold extremities as well as convulsions, were negatively associated with bacteremia and seem to suggest malaria as a primary diagnosis.

In general, children below the age of five years with a low parasite count of <10,000/l are at higher risk for bacteremic co-infection than others.

When stratifying for high parasite count of >10,000/μl blood, clinical signs of respiratory infection like coughing, drawing-in of chest wall and respiratory distress gain even more in importance. Dehydration and tenting of skin fold are also strongly associated with systemic blood stream infection. Skin infections were not detected very often, but in cases of any skin abnormalities of the severely ill child, the likelihood of a bacteremic co-infection was higher than without pathologic skin aspects. In this subgroup, high fever was not positively associated with bacteremia.

In order to identify the most influential clinical predictors for co-infection with bacteremia, we applied parsimonious stepwise multivariate regression analysis on the subgroup presenting with high parasitemia. We could select coughing, dehydration and splenomegaly as the three factors with strongest association. In the subgroup of low parasitemia, cellular blood parameters like severe anemia and leukocytosis as well as vomiting were highly associated. Even with a moderate sensitivity of >60%, only low positive predictive values <40% can be obtained due to low prevalence of bacteremia (Tables 4 and 5).

A combination of two or all clinical predictors in terms of an algorithm could not increase the positive or negative predictive value of single variables.

## Discussion

Differentiation of causative infectious agents in children with severe disease in malaria endemic areas in sub-Saharan Africa is a challenge for clinicians. Many efforts have been made to detect clinical or laboratory discriminators powerful and feasible enough to support clinicians in the diagnosis finding process [1,2,5,19,20,21].

Unlike frequently quoted related studies from Tanzania and Kenya [1,2], we did not apply currently recommended guidelines on our data to analyze their validity, but tested a comprehensive set of clinical, socio-economic, history and laboratory data for their association with and prediction of bacteremia in children with malaria parasitemia. With this approach, we intended to find indicators that might complement existing clinical algorithms. In a recent prospective study from Tanzania, it has been demonstrated that in a population exposed to intense *P*. *falciparum* transmission, WHO guidelines failed to identify almost one-third of invasive bacterial infections [1]. Because of overlapping clinical symptoms, sensitivity for clinical diagnosis of bacteremia was reduced with increasing *P*. *falciparum* density from 70% in slide negative children to 53% in children with a parasite count of more than 50,000 parasites/μl. Knowledge of parasite count might therefore be critical for a diagnostic algorithm to screen for an additional bacteremia. In our study design, we referred to this hypothesis and generated different algorithms for high (>10,000/μl) and low parasite counts.

Out of 771 children with *P*. *falciparum* parasitemia, 6% were co-infected with invasive bacteria. Non-typhoidal salmonellae and *Streptococcus* spp. were the most frequent isolates irrespective of a positive or negative malaria count. This was twice as high as in observations of a Kenyan study, where 3,0% of children had invasive bacterial infections and positive malarial slides [2]. Direct comparability of the results is limited since children in Kenya were younger (median 17 months) and children with bacteremia and meningitis were included in the group of invasive bacterial infections. In general, co-infection is more commonly reported in areas of high malaria transmission than in areas with low transmission intensity [22].

### Clinical factors associated with bacteremia in parasitemic children

Results from Tanzania [1] identified “severe anemia” (hemoglobin <5 g/dl), “prostration” and “current fever >38°C” to increase sensitivity of current WHO guidelines, resulting in a significant increase of detection of bacteremic children. Our findings confirm a positive association of severe anemia (<8 g/dl) with bacteremia in the group of all children below the age of 15 years. In this special setting of co-infection, prostration and fever was not identified as an important distinguishing factor, but fever and especially high fever was obviously significantly positively associated with bacteremia, when not stratified for parasitemia. Prostration did not show a significant predictive value in our models. In conclusion, considering our results, severe anemia should further be investigated as a valuable factor of identifying bacterial co-infection in high transmission areas for malaria. Additional factors that should be considered in algorithms to identify a bacterial co-infection are coughing, dehydration, splenomegaly and leukocytosis as well as a developmental delay of the child (Table 2).

### Positive clinical predictors for bacteremia in children with high parasite count

Asymptomatic parasitemia in children is frequent in high transmission areas. Data from Uganda suggests an occurrence of 30% in school children [23]. After acquiring semi-immunity, asymptomatic infections account for 12–52%, depending on the diagnostic method [24]. In this study, the subgroup of children with high parasitemia is large, arguing against co-existing asymptomatic parasitemia.

According to stepwise multivariate analysis, dehydration, splenomegaly and coughing show strong association with invasive bacterial co-infection in children with high parasite counts (Table 4). Due to low prevalence of bacteremia, even the best positive predictive values detected, namely for dehydration, are only 33% (Table 5). Indeed, signs of dehydration, e.g. tenting of the skin, are strongly associated with bacteremia in highly parasitemic children (Table 3). Dehydration can be the result of limited rehydration due to the inability to breastfeed or drink, due to diarrhea or generalized reactions like fever or shock. Diarrhea and vomiting are typical symptoms for gastroenteritis with NTS but can also occur in cases with NTS bacteremia [25].

Clinical presentation of invasive NTS infection is diverse. Recently published review data on invasive non-typhoidal salmonella disease describes clinical presentation with fever [25], hepatosplenomegaly and respiratory symptoms [26] as common features, whereas symptoms of enterocolitis are often absent [27]. The data presented here can statistically reproduce this finding, since coughing (OR = 9.0) and splenomegaly (OR = 8.8) in parasitemic children are strongly associated with bacteremia. After all, in our model, high parasite counts go along with increased mortality in children without co-infection as well as in the only case with additional bacteremia, stressing the importance of this parameter.

### Positive clinical predictors for bacteremia in children with low parasite count

In multivariate regression analysis, laboratory factors that are typically altered in invasive bacteremic infection, like leukocytosis and low hemoglobin, can prevail despite similar symptom complexes occurring in clinical malaria. Additionally, vomiting was detected as a major positive clinical predictor of bacteremia in children with low parasite count. Besides fever, vomiting is a commonly reported symptom (90%) of malaria in children [28]. Recent data from Ghana describes a history of vomiting in 41% of children with parasites detected in thick smear. In our study collective, half of highly parasitemic children were reported by guardians to have vomited previously, and only 36% of those with low parasite count were affected. The percentage of children vomiting in the bacteremic group was similarly high with 35%. However, vomiting seems to be associated with bacteremia and bacterial co-infection, although it is known as a clinical feature linked to parasitemia.

### Impaired microcirculation—a valuable discriminating factor?

In a bivariate regression model, prolonged capillary refill time >2 sec was significantly negatively associated with systemic bacterial blood stream infections independent of level of parasitemia. In contrast, cold extremities, another clinical sign for an impaired cardiovascular system, indicated a bacterial co-infection in children with high parasitemia. It has been reported that a prolonged capillary refill time is highly associated with severe malaria [29]. Nevertheless, signs of cardiovascular dysfunction (prolonged capillary refill >5 sec; core to peripheral temperature gap >3°C) belong to the organ dysfunction criteria of the latest definition of pediatric severe sepsis and septic shock criteria and might rather be a sign of severe disease [17].

Many epidemiological, clinical and molecular studies suggest a positive association between malaria and invasive NTS bacteremia, reasoning that invasive NTS infections could be reduced by eliminating malaria [30,31,32]. Our findings may support this theory, since usage of mosquito nets was significantly associated with a lower occurrence of bacteremia. Another explanation for this strong association could be confounding through higher hygiene standards and health education in these families. This is in accordance with our finding that a high social-economic background allowing a water closet and proper cooking facilities in the house are negatively associated with bacterial co-infections. Young children who have not been weaned, but are exclusively breastfed, show significantly less invasive bacterial infections in this study group compared to children receiving other liquids or solid food, indicating oral transmission through contaminated food. In general, wealthy families seem to have the financial opportunities to seek professional medical help more often, represented in the association of invasive bacterial disease and a high level of education (see S1 Table). Nevertheless, these results need to be interpreted carefully, since they may be confounded.

### Validity and generalizability of results

In this study, a large set of socio-economic, clinical and laboratory parameters was analyzed including basic clinical WHO criteria of the manual “management of the child with a serious infection or severe malnutrition: guidelines for care at first-referral level in developing countries”. The focus of this study was to investigate clinical parameters for the use under limited conditions. However, additional laboratory diagnostic suggested by WHO (e.g. bacteriological culture carried out on the urine, lumbar puncture [16]) as well as HIV co-infection were not evaluated and may be considered in further studies. Although the total number of patients in this study was large, the absolute number of children with malaria and a positive blood culture was too small to achieve sufficient statistical power for further association analyses and stratification for specific bacterial pathogens. The study was linked to the hospital routine health care and missing data points were not completely preventable. Missing admissions at night and weekends might have led to a selection bias with underestimation of severe diseases. Future projects should consider these obstacles in terms of study design and data management. Some variables, such as assessment of “cold extremities,” sometimes underlie subjective interpretation. Assessment of socio-economic and socio-demographic data was prone to reporting bias.

## Conclusions

The results presented here demonstrate the limitation of clinical algorithms to clinically determine bacterial co-infections in children with malarial parasitemia. Best clinical indicators are dependent on the parasite level, and even with an acceptable sensitivity of >60%, only low positive predictive values can be obtained due to the low prevalence of bacteremia. In children with parasite counts >10,000/μl presenting to a rural hospital in a malaria endemic area of Ghana, best indicators for bacterial co-infection were dehydration, splenomegaly and coughing. In children with lower parasite counts, best indicators were severe anemia, leukocytosis and vomiting. These symptom constellations indicate that presumptive antimicrobial treatment, e.g. with ceftriaxone, would be beneficial.

Nevertheless, the combination of clinical and demographic indicators cannot replace thorough microbiological diagnosis. Rapid testing on the basis of biochemical markers for distinguishing parasitemia and bacteremia should be further optimized for countries with limited resources.

## Supporting Information

S1 Table Bivariate regression analysis of all variables in parasitemic children <15 years. (DOCX)

## References

[1] Nadjm B, Amos B, Mtove G, Ostermann J, Chonya S, Wangai H, et al WHO guidelines for antimicrobial treatment in children admitted to hospital in an area of intense Plasmodium falciparum transmission: prospective study. BMJ. 2010; 340: c1350 10.1136/bmj.c1350 20354024 PMC2847687

[2] Berkley JA, Maitland K, Mwangi I, Ngetsa C, Mwarumba S, Lowe BS, et al Use of clinical syndromes to target antibiotic prescribing in seriously ill children in malaria endemic area: observational study. BMJ. 2005; 330: 995 15797893 10.1136/bmj.38408.471991.8F PMC557145

[3] Evans JA, Adusei A, Timmann C, May J, Mack D, Agbenyega T, et al High mortality of infant bacteraemia clinically indistinguishable from severe malaria. QJM. 2004; 97: 591–597. 15317928 10.1093/qjmed/hch093

[4] Amexo M, Tolhurst R, Barnish G, Bates I. Malaria misdiagnosis: effects on the poor and vulnerable. Lancet. 2004; 364: 1896–1898. 15555670 10.1016/S0140-6736(04)17446-1

[5] Reyburn H, Mbatia R, Drakeley C, Carneiro I, Mwakasungula E, Mwerinde O, et al Overdiagnosis of malaria in patients with severe febrile illness in Tanzania: a prospective study. BMJ. 2004; 329: 1212 15542534 10.1136/bmj.38251.658229.55 PMC529364

[6] Kahama-Maro J, D'Acremont V, Mtasiwa D, Genton B, Lengeler C. Low quality of routine microscopy for malaria at different levels of the health system in Dar es Salaam. Malar J. 2011; 10: 332 10.1186/1475-2875-10-332 22047131 PMC3217957

[7] Nielsen MV, Sarpong N, Krumkamp R, Dekker D, Loag W, Amemasor S, et al Incidence and Characteristics of Bacteremia among Children in Rural Ghana. PLoS One. 2012; 7: e44063 10.1371/journal.pone.0044063 22970162 PMC3438186

[8] WHO: Agogo Presbyterian Hospital, Ghana. 2011; Available: http://www.who.int/buruli/events/agogo_hospital/en/index.html Accessed 2015 February 14.

[9] Ghana. Cities & Urban Localities. Census Data of the year 2000. 2011; Available: http://www.citypopulation.de/Ghana-Cities.html Accessed 2015 February 14.

[10] WHO. World malaria report 2009. 2009; Available: http://www.who.int/malaria/publications/country-profiles/2009/mal2009_ghana_0022.pdf Accessed 2015 February 14.

[11] Browne EN, Frimpong E, Sievertsen J, Hagen J, Hamelmann C, Dietz K, et al Malariometric update for the rainforest and savanna of Ashanti region, Ghana. Ann Trop Med Parasitol. 2000; 94: 15–22. 10723520

[12] Krefis AC, Schwarz NG, Kruger A, Fobil J, Nkrumah B, Acquah S, et al Modeling the relationship between precipitation and malaria incidence in children from a holoendemic area in Ghana. Am J Trop Med Hyg. 2011; 84: 285–291. 10.4269/ajtmh.2011.10-0381 21292900 PMC3029183

[13] Sarpong N, Loag W, Fobil J, Meyer CG, Adu-Sarkodie Y, May J, et al National health insurance coverage and socio-economic status in a rural district of Ghana. Trop Med Int Health. 2010; 15: 191–197. 10.1111/j.1365-3156.2009.02439.x 19961565

[14] CIA: CIA factbook 2012. 2012; Available: https://www.cia.gov/library/publications/the-world-factbook/geos/gh.html Accessed 2015 February 14.

[15] Renner LA, Newman MJ, Ahadzie L, Antwi-Agyei KO, Eshetu M. Introduction of Haemophilus influenzae type B conjugate vaccine into routine immunization in Ghana and its impact on bacterial meningitis in children younger than five years. Pediatr Infect Dis J. 2007; 26: 356–358. 17414404 10.1097/01.inf.0000258693.19247.8e

[16] WHO Management of the child with a serious infection or severe malnutrition: guidelines for care at the first-referral level in developing countries. 2000; Available: http://whqlibdoc.who.int/hq/2000/WHO_FCH_CAH_00.1.pdf Accessed 2015 February 14.

[17] Goldstein B, Giroir B, Randolph A. International pediatric sepsis consensus conference: definitions for sepsis and organ dysfunction in pediatrics. Pediatric critical care medicine: a journal of the Society of Critical Care Medicine and the World Federation of Pediatric Intensive and Critical Care Societies. 2005; 6: 2–8. 10.1097/01.PCC.0000149131.72248.E6 15636651

[18] Krefis AC, Schwarz NG, Nkrumah B, Acquah S, Loag W, Sarpong N, et al Principal component analysis of socioeconomic factors and their association with malaria in children from the Ashanti Region, Ghana. Malar J. 2010; 9: 201 10.1186/1475-2875-9-201 20626839 PMC2914064

[19] Lathia TB, Joshi R. Can hematological parameters discriminate malaria from nonmalarious acute febrile illness in the tropics? Indian J Med Sci. 2004; 58: 239–244. 15226575

[20] Planche T, Agbenyega T, Bedu-Addo G, Ansong D, Owusu-Ofori A, Micah F, et al A prospective comparison of malaria with other severe diseases in African children: prognosis and optimization of management. Clin Infect Dis. 2003; 37: 890–897. 13130399 10.1086/377536

[21] Ayoola OO, Adeyemo AA, Osinusi K. Predictors of bacteraemia among febrile infants in Ibadan, Nigeria. J Health Popul Nutr. 2002; 20: 223–229. 12430758

[22] Church J, Maitland K. Invasive bacterial co-infection in African children with Plasmodium falciparum malaria: a systematic review. BMC medicine. 2014; 12: 31 10.1186/1741-7015-12-31 24548672 PMC3928319

[23] Nankabirwa J, Wandera B, Kiwanuka N, Staedke SG, Kamya MR, Brooker SJ. Asymptomatic Plasmodium infection and cognition among primary schoolchildren in a high malaria transmission setting in Uganda. The American journal of tropical medicine and hygiene. 2013; 88: 1102–1108. 10.4269/ajtmh.12-0633 23589533 PMC3752809

[24] Dal-Bianco MP, Koster KB, Kombila UD, Kun JF, Grobusch MP, Ngoma GM, et al High prevalence of asymptomatic Plasmodium falciparum infection in Gabonese adults. The American journal of tropical medicine and hygiene. 2007; 77: 939–942. 17984357

[25] Kariuki S, Revathi G, Kariuki N, Kiiru J, Mwituria J, Hart CA. Characterisation of community acquired non-typhoidal Salmonella from bacteraemia and diarrhoeal infections in children admitted to hospital in Nairobi, Kenya. BMC microbiology. 2006; 6: 101 17173674 10.1186/1471-2180-6-101 PMC1764016

[26] Schwarz NG, Sarpong N, Hunger F, Marks F, Acquah SE, Agyekum A, et al Systemic bacteraemia in children presenting with clinical pneumonia and the impact of non-typhoid salmonella (NTS). BMC infectious diseases. 2010; 10: 319 10.1186/1471-2334-10-319 21050455 PMC2991321

[27] Feasey NA, Dougan G, Kingsley RA, Heyderman RS, Gordon MA. Invasive non-typhoidal salmonella disease: an emerging and neglected tropical disease in Africa. Lancet. 2012; 379: 2489–2499. 10.1016/S0140-6736(11)61752-2 22587967 PMC3402672

[28] Choge JK, Magak NG, Akhwale W, Koech J, Ngeiywa MM, Oyoo-Okoth E, et al Symptomatic malaria diagnosis overestimate malaria prevalence, but underestimate anaemia burdens in children: results of a follow up study in Kenya. BMC public health. 2014; 14: 332 10.1186/1471-2458-14-332 24712340 PMC3996101

[29] Evans JA, May J, Ansong D, Antwi S, Asafo-Adjei E, Nguah SB, et al Capillary refill time as an independent prognostic indicator in severe and complicated malaria. The Journal of pediatrics. 2006; 149: 676–681. 17095342 10.1016/j.jpeds.2006.07.040

[30] Scott JA, Berkley JA, Mwangi I, Ochola L, Uyoga S, Macharia A, et al Relation between falciparum malaria and bacteraemia in Kenyan children: a population-based, case-control study and a longitudinal study. Lancet. 2011; 378: 1316–1323. 10.1016/S0140-6736(11)60888-X 21903251 PMC3192903

[31] Maltha J, Guiraud I, Kabore B, Lompo P, Ley B, Bottieau E, et al Frequency of severe malaria and invasive bacterial infections among children admitted to a rural hospital in Burkina Faso. PLoS One. 2014; 9: e89103 10.1371/journal.pone.0089103 24551225 PMC3925230

[32] Lokken KL, Mooney JP, Butler BP, Xavier MN, Chau JY, Schaltenberg N, et al Malaria Parasite Infection Compromises Control of Concurrent Systemic Non-typhoidal Salmonella Infection via IL-10-Mediated Alteration of Myeloid Cell Function. PLoS pathogens. 2014; 10: e1004049 10.1371/journal.ppat.1004049 24787713 PMC4006898

