# Supplementary material for: Clinical Indicators for Bacterial Co-Infection in Ghanaian Children with P. falciparum Infection
Source: PLoS One. 2015 Apr 9;10(4):e0122139. doi: 10.1371/journal.pone.0122139 (PMC4391931; doi:10.1371/journal.pone.0122139)
Supplement: S1 Table — (DOCX) [file pone.0122139.s001.docx]

Table S1 Bivariate regression analysis of all variables in parasitemic children <15 years.

|  |  | Bacteremia (n=771) | | | |
| --- | --- | --- | --- | --- | --- |
|  |  | n | | p-value | OR (CI) |
|  |  | No | Yes (%) |  |  |
| Sex | Male | 153 | 14 (8.4) |  | 1 |
|  | Female | 168 | 21 (11.1) | 0.72 | 0.90 (0.49-1.63) |
| Age | <5 years | 575 | 42 (6.8) |  | 1 |
|  | ≥5 years | 150 | 4 (2.6) | 0.06 | 0.37 (0.13-1.03) |
| Vaccination status^1^ | None/Incomplete | 28 | 1 (3.5) |  | 1 |
|  | Complete | 674 | 45 (6.3) | 0.54 | 1.87 (0.25-14.05) |
| Weight-for-age  Z-score<-2 (Underweight)^2^ | No | 502 | 32 (6.0) |  | 1 |
|  | Yes | 104 | 11 (9.6) | 0.17 | 1.66 (0.81-3.40) |
| Weight-for-length  Z-score<-2 (Wasting)^2^ | No | 219 | 21 (8.8) |  | 1 |
|  | Yes | 53 | 7 (11.7) | 0.49 | 1.38 (0.56-3.41) |
| Length-for-age  Z-score<-2 (Stunting)^2^ | No | 207 | 22 (9.6) |  | 1 |
|  | Yes | 65 | 7 (9.7) | 0.98 | 1.01 (0.41-2.48) |
| Developmental delay^3^ | No | 129 | 2 (1.5) |  | 1 |
|  | Yes | 12 | 2 (14.3) | 0.027 | 10.18 (1.39-79.53) |
| Exclusive breastfeeding | No | 111 | 14 (11.2) |  | 1 |
|  | Yes | 61 | 1 (1.6) | 0.051 | 0.13 (0.02-1.01) |
| Intake of antimalarials | No | 636 | 37 (5.5) |  | 1 |
|  | Yes | 73 | 7 (8.8) | 0.25 | 1.65 (0.71-3.83) |
| Intake of antibiotics | No | 654 | 43 (6.2) |  | 1 |
|  | Yes | 64 | 1 (1.5) | 0.16 | 0.24 (0.03-1.75) |
| Intake of antihelmints | No | 460 | 27 (5.5) |  | 1 |
|  | Yes | 209 | 13 (5.9) | 0.87 | 1.06 (0.54-2.09) |
| Intake of paracetamol | No | 190 | 10 (5.0) |  | 1 |
|  | Yes | 529 | 35 (6.2) | 0.54 | 1.26 (0.61-2.59) |
| History of fever | No | 53 | 1 (1.9) |  | 1 |
|  | Yes | 670 | 44 (6.2) | 0.22 | 3.48 (0.47-25.76) |
| History of cough | No | 526 | 25 (4.5) |  | 1 |
|  | Yes | 196 | 20 (9.3) | 0.014 | 2.15 (1.17-3.95) |
| History of vomiting | No | 406 | 31 (7.1) |  | 1 |
|  | Yes | 316 | 14 (4.2) | 0.10 | 0.58 (0.30-1.11) |
| History of diarrhea | No | 611 | 36 (5.6) |  | 1 |
|  | Yes | 111 | 9 (7.5) | 0.41 | 1.38 (0.64-2.94) |
| Skin abnormalities | No | 289 | 31 (9.7) |  | 1 |
|  | Yes | 14 | 3 (17.7) | 0.30 | 2.00 (0.54-7.34) |
| Skin rash | No | 290 | 33 (10.2) |  | 1 |
|  | Yes | 11 | 1 (8.3) | 0.83 | 0.80 (0.10-6.39) |
| Skin depigmentation | No | 294 | 34 (10.4) |  | 1 |
|  | Yes | 9 | 0 (0.0) | - | - |
| Lymphadenopathy | No | 684 | 36 (5.0) |  | 1 |
|  | Yes | 3 | 0 (0.0) | - | - |
| Body temperature (axillary) | Normothermia (35.0-37.4°C) | 141 | 9 (6.0) |  | 1 |
|  | Hypothermia  (<35.0°C) | 2 | 0 (0.0) | - | - |
|  | Hyperthermia  (≥37.5°C) | 582 | 37 (6.0) | 0.10 | 1.00 (0.47-2.11) |
| High fever ≥ 38.5°C |  | 336 | 21 (5.9) |  | 1 |
|  |  | 389 | 25 (6.0) | 0.93 | 1.03 (0.57-1.87) |
| Fever > 5 days | No | 152 | 31 (16.9) |  | 1 |
|  | Yes | 13 | 1 (7.1) | 0.36 | 0.38 (0.05-2.99) |
| Stiff neck | No | 708 | 45 (6.0) |  | 1 |
|  | Yes | 12 | 1 (7.7) | 0.80 | 1.31 (0.17-10.31) |
| Bulging of fontanel | No | 484 | 26 (5.1) |  | 1 |
|  | Yes | 14 | 0 (0.0) | - | - |
| Signs of meningitis | No | 479 | 26 (5.2) |  | 1 |
|  | Yes | 23 | 1 (4.2) | 0.83 | 0.80 (0.10-6.16) |
| Able to breastfeed | No | 403 | 28 (6.5) |  | 1 |
|  | Yes | 219 | 6 (2.7) | 0.042 | 0.39 (0.16-0.97) |
| Weakness | No | 474 | 23 (4.6) |  | 1 |
|  | Yes | 211 | 17 (7.5) | 0.13 | 1.66 (0.87-3.17) |
| Unconsciousness | No | 632 | 36 (5.4) |  | 1 |
|  | Yes | 48 | 3 (5.9) | 0.96 | 1.03 (0.30-3.50) |
| Restlessness | No | 666 | 37 (5.3) |  | 1 |
|  | Yes | 15 | 1 (6.3) | 0.86 | 1.2 (0.15-9.33) |
| Convulsions | No | 476 | 39 (7.6) |  | 1 |
|  | Yes | 224 | 7 (3.0) | 0.019 | 0.38 (0.17-0.85) |
| Blantyre come scale ≤4 | No | 640 | 39 (5.7) |  | 1 |
|  | Yes | 56 | 6 (9.7) | 0.22 | 1.76 (0.71-4.33) |
| Cardiac fatigue^4^ | No | 260 | 32 (11.0) |  | 1 |
|  | Yes | 34 | 2 (5.6) | 0.33 | 0.48 (0.11-2.08) |
| Cyanosis | No | 662 | 35 (5.0) |  | 1 |
|  | Yes | 3 | 0 (0.0) | - | - |
| Heartrate^5^ | Normocardia | 265 | 12 (4.3) |  | 1 |
|  | Tachycardia | 318 | 23 (6.7) | 0.20 | 1.60 (0.78-3.27) |
|  | Bradycardia | 37 | 1 (2.6) | 0.63 | 0.60 (0.07-4.72) |
| Circulation impaired^6^ | No | 42 | 7 (14.3) |  | 1 |
|  | Yes | 644 | 29 (4.3) | 0.004 | 0.27 (0.11-0.65) |
| Cold extremities | No | 686 | 35 (4.9) |  |  |
|  | Yes | 6 | 1 (14.3) | 0.28 | 3.27 (0.38-27.88) |
| Capillary refill time ≥2 | No | 144 | 23 (13.8) |  |  |
|  | Yes | 536 | 12 (2.2) | 0.000 | 0.14 (0.07-0.29) |
| Prostration | No | 534 | 33 (5.8) |  | 1 |
|  | Yes | 161 | 13 (7.5) | 0.43 | 1.31 (0.67-2.54) |
| Palmar pallor | No | 292 | 11 (3.6) |  | 1 |
|  | Yes | 401 | 25 (5.9) | 0.17 | 1.65 (0.80-3.42) |
| Poor general conditions | No | 301 | 16 |  | 1 |
|  | Yes | 360 | 22 (5.8) | 0.68 | 1.15 (0.59-2.23) |
| Cough | No | 477 | 23 (4.6) |  | 1 |
|  | Yes | 245 | 22 (8.2) | 0.04 | 1.86 (1.02-3.41) |
| Difficulties breathing | No | 692 | 43 (5.9) |  | 1 |
|  | Yes | 30 | 2 (6.3) | 0.93 | 1.07 (0.25-4.64) |
| Fast breathing | No | 650 | 39 (5.7) |  | 1 |
|  | Yes | 49 | 6 (10.9) | 0.12 | 2.04 (0.82-5.06) |
| Breathing deeply | No | 696 | 45 (6.1) |  | 1 |
|  | Yes | 25 | 1 (3.9) | 0.64 | 0.62 (0.08-4.67) |
| Drawing-in of chest wall | No | 687 | 44 (6.0) |  | 1 |
|  | Yes | 34 | 2 (5.6) | 0.91 | 0.92 (0.21-3.95) |
| Stridor | No | 688 | 36 (5.0) |  | 1 |
|  | Yes | 2 | 0 (0.0) | - | - |
| Respiratory distress | No | 654 | 32 (4.7) |  | 1 |
|  | Yes | 35 | 3 (7.9) | 0.41 | 1.43 (0.61-3.35) |
| Breathing rate^7^ | Normal | 300 | 14 (4.5) |  | 1 |
|  | Fast | 266 | 16 (5.7) | 0.50 | 1.29 (0.62-2.69) |
|  | Slowly | 50 | 3 (5.7) | 0.70 | 1.29 (0.36-4.64) |
| Diarrhea | No | 580 | 35 (5.7) |  | 1 |
|  | Yes | 142 | 9 (6.0) | 0.90 | 1.05 (0.49-2.23) |
| Heavy diarrhoea^8^ | No | 86 | 5 (5.5) |  | 1 |
|  | Yes | 42 | 4 (8.7) | 0.48 | 1.64 (0.42-6.42) |
| Watery stool | No | 143 | 22 (13.3) |  | 1 |
|  | Yes | 121 | 9 (6.9) | 0.08 | 0.48 (0.21-1.09) |
| Mucous stool | No | 64 | 5 (7.3) |  | 1 |
|  | Yes | 78 | 4 (4.9) | 0.54 | 0.66 (0.17-2.55) |
| Bloody stool | No | 138 | 8 (5.5) |  | 1 |
|  | Yes | 4 | 1 (20.0) | 0.21 | 4.31 (0.43-43.20) |
| Vomiting | No | 388 | 22 (5.4) |  | 1 |
|  | Yes | 334 | 23 (6.4) | 0.53 | 1.21 (0.66-2.22) |
| Vomiting everything | No | 192 | 10 (5.0) |  | 1 |
|  | Yes | 139 | 12 (8.0) | 0.25 | 1.66 (0.70-3.95) |
| Thirsty drinking | No | 303 | 30 (9.0) |  | 1 |
|  | Yes | 418 | 15 (3.5) | 0.002 | 0.36 (0.19-0.69) |
| Sunken eyes | No | 686 | 36 (5.0) |  | 1 |
|  | Yes | 6 | 0 (0.0) | - | - |
| Apathy | No | 668 | 36 (5.1) |  | 1 |
|  | Yes | 24 | 0 (0.0) | - | - |
| Tenting of skin | No | 689 | 35 (4.8) |  | 1 |
|  | Yes | 3 | 1 (25.0) | 0.11 | 6.56 (0.67-64.70) |
| Ascites | No | 691 | 35 (4.8) |  | 1 |
|  | Yes | 1 | 0 (0.0) | - | - |
| Splenomegaly | No | 497 | 11 (2.2) |  | 1 |
|  | Yes | 58 | 7 (10.8) | 0.001 | 5.45 (2.03-14.61) |
| Hepatomegaly | No | 501 | 12 (2.3) |  | 1 |
|  | Yes | 50 | 2 (3.9) | 0.51 | 1.67 (0.36-7.67) |
| Dehydration^9^ | No | 656 | 32 (4.7) |  | 1 |
|  | Yes | 12 | 3 (20.0) | 0.015 | 5.13 (1.38-19.07) |
| Acute malnutrition | No | 465 | 28 (5.7) |  | 1 |
|  | Yes | 127 | 11 (8.0) | 0.33 | 1.44 (0.70-2.97) |
| Wasted^10^ | No | 709 | 46 (6.1) |  |  |
|  | Yes | 14 | 0 (0.0) | - | - |
| Malnourished | No | 675 | 44 (6.1) |  | 1 |
|  | Yes | 44 | 1 (2.2 | 0.30 | 0.35 (0.47-2.59) |
| Poor feeding | No | 268 | 16 (5.6) |  | 1 |
|  | Yes | 425 | 28 (6.2) | 0.76 | 1.10 (0.59-2.08) |
| Reduced skin turgor | No | 159 | 28 (15.0) |  | 1 |
|  | Yes | 0 | 1 (100.0) | - | - |
| Jaundice | No | 624 | 31 (4.7) |  | 1 |
|  | Yes | 68 | 5 (6.9) | 0.43 | 1.48 (0.56-3.93) |
| Edema of feed | No | 685 | 36 (5.0) |  | 1 |
|  | Yes | 6 | 0 (0.0) | - | - |
| Kwashiorkor | No | 680 | 41 (5.7) |  | 1 |
|  | Yes | 4 | 0 (0.0) | - | - |
| Ear pain | No | 297 | 33 (10.0) |  | 1 |
|  | Yes | 6 | 1 (14.3) | 0.71 | 1.50 (0.18-12.84) |
| Ear discharge | No | 302 | 34 (10.1) |  | 1 |
|  | Yes | 1 | 0 (0.0) | - | - |
| Any ear symptoms | No | 247 | 27 (9.9) |  | 1 |
|  | Yes | 6 | 1 (14.3) | 0.70 | 1.52 (0.10-13.14) |
| Throat pain | No | 303 | 33 (9.8) |  | 1 |
|  | Yes | 0 | 1 (100.0) | - | - |
| Ear pus | No | 253 | 28 (10.0) |  | 1 |
|  | Yes | 0 | 0 (0.0) | - | - |
| Blocked nose | No | 612 | 41 (6.3) |  | 1 |
|  | Yes | 87 | 4 (4.4) | 0.48 | 0.69 (0.24-1.96) |
| Rhinorrhea | No | 478 | 33 (6.5) |  | 1 |
|  | Yes | 220 | 12 (5.2) | 0.50 | 0.79 (0.40-1.56) |
| Rhinitis | No | 456 | 31 (6.4) |  | 1 |
|  | Yes | 242 | 14 (5.5) | 0.63 | 0.85 (0.44-1.63) |
| White blood cell count^11^ | Normocytic | 379 | 15 (3.8) |  | 1 |
|  | Leukocytosis | 266 | 28 (9.5) | 0.003 | 2.66 (1.39-5.08) |
|  | Leukopenia | 77 | 3 (3.8) | 0.98 | 0.98 (0.28-3.48) |
| Anemia^12^ | No | 98 | 6 (5.8) |  | 1 |
|  | Yes | 625 | 40 (6.0 | 0.92 | 1.05 (0.43-2.53) |
| Severe anemia <8 mg/dl | No | 442 | 18 (3.9) |  | 1 |
|  | Yes | 281 | 28 (9.1) | 0.004 | 2.45 (1.33-4.51) |
| Parasitemia | <10,000/µl | 153 | 15 (8.9) |  | 1 |
|  | >10,000/µl | 441 | 17 (3.7) | 0.01 | 0.39 (0.19-0.81) |
| From Agogo | No | 392 | 24 (5.8) |  | 1 |
|  | Yes | 333 | 22 (6.2) | 0.80 | 1.08 (0.59-1.96) |
| Born in hospital | No | 173 | 4 (2.3) |  | 1 |
|  | Yes | 380 | 15 (3.8) | 0.35 | 1.70 (0.56-5.22) |
| Religion | Christian | 527 | 32 (5.7) |  | 1 |
|  | Moslem | 83 | 2 (2.4) | 0.21 | 0.40 (0.09-1.69) |
|  | Other | 32 | 2 (5.9) | 0.97 | 1.03 (0.24-4.49) |
| Traditional healer | No | 82 | 17 (17.2) |  | 1 |
|  | Yes | 17 | 6 (26.1) | 0.33 | 1.70 (0.59-4.95) |
| Ethnicity | Akan and Others | 536 | 31 (5.5) |  | 1 |
|  | Northerners | 188 | 15 (7.4) | 0.32 | 1.38 (0.73-2.61) |
| Number children <5^13^ | No | 157 | 9 (5.4) |  | 1 |
|  | Yes | 525 | 31 (5.6) | 0.94 | 1.03 (0.48-2.21) |
| Mother ≥ 30 years | No | 326 | 25 (7.1) |  | 1 |
|  | Yes | 334 | 21 (5.9) | 0.82 | 0.52 (0.45-1.49) |
| Mosquito net | No | 523 | 33 (5.9) |  | 1 |
|  | Bed/ window net | 119 | 1 (0.1) | 0.05 | 0.13 (0.02-0.98) |
| Income management | Difficult | 508 | 29 (5.4) |  | 1 |
|  | Not difficult | 132 | 7 (5.0) | 0.87 | 0.93 (0.40-2.17) |
| Washing their hands | Not before meals | 50 | 8 (13.8) |  |  |
|  | Before meals | 592 | 27 (4.4) | 0.003 | 0.29 (0.12-0.66) |
| Cooking | Outside | 40 | 5 (11.1) |  | 1 |
|  | Kitchen | 134 | 19 (12.4) | 0.81 | 1.13 (0.40-3.23) |
| People eating from one jar | 1-4 | 173 | 22 (11.3) |  |  |
|  | 5-12 | 44 | 9 (17.0) | 0.27 | 1.60 (0.69-3.74) |
| Electricity | No | 259 | 16 (5.8) |  | 1 |
|  | Yes | 383 | 20 (5.0) | 0.59 | 0.73 (0.23-2.29) |
| Food storage | Heating/ smoking | 82 | 21 (20.4) |  | 1 |
|  | Freezing | 16 | 2 (11.1) | 0.36 | 0.49 (0.10-2.29) |
| Water supply | River/Well | 103 | 10 (8.9) |  | 1 |
|  | Tap/pipe | 539 | 26 (4.6) | 0.07 | 0.50 (0.23-1.06) |
| Toilet in house | No | 312 | 25 (7.4) |  | 1 |
|  | Yes | 330 | 10 (2.9) | 0.29 | 0.52 (0.15-1.77) |
| House type | Cement/Brick | 473 | 26 (5.2) |  | 1 |
|  | Wood/ Mud | 169 | 10 (5.6) | 0.85 | 1.08 (0.51-2.28) |
| Relative abroad | No | 185 | 28 (13.2) |  | 1 |
|  | Yes | 31 | 3 (8.8) | 0.48 | 0.64 (0.18-2.23) |
| Mother literate | No | 175 | 24 (12.1) |  | 1 |
|  | Yes | 41 | 7 (14.6) | 0.64 | 1.24 (0.50-3.01) |
| Father literate | No | 85 | 9 (9.6) |  | 1 |
|  | Yes | 130 | 22 (14.5) | 0.26 | 1.60 (0.70-3.64) |
| Mother employment | No | 50 | 4 (7.4) |  | 1 |
|  | Yes | 591 | 32 (5.1) | 0.48 | 0.68 (0.23-1.99) |
| Father employment | No | 28 | 1 (3.5) |  | 1 |
|  | Yes | 599 | 35 (5.5) | 0.63 | 1.64 (0.22-12.38) |
| Health insurance | No | 236 | 13 (5.2) |  | 1 |
|  | Yes | 406 | 23 (5.4) | 0.38 | 0.60 (0.19-1.88) |
| Socioeconomic status | Low | 218 | 17 (7.2) |  | 1 |
|  | Medium | 216 | 11 (4.9) | 0.29 | 0.65 (0.30-1.43) |
|  | High | 254 | 13 (4.9) | 0.27 | 0.66 (0.31-1.38) |

^1^ Vaccination against tuberculosis, diphtheria, pertussis, tetanus, measles and yellow fever

^2^ Underweight (weight-for-age), wasting (weight-for-height) and stunting (height/length-for-age) using 2006 WHO child growth standards. Cut-off points were Z-scores of ±2 as suggested by WHO in 1997.

^3^ Children, who cannot hold their head at the age of three months, roll over at the age of 6 months, sit unsupported at the age of 9 months, stand unsupported at the age of 12 months or walk single steps at the age of 18 months

^4^ Clinical signs of heart failure

^5^ Bradycardia defined as <110 beats per minute (bpm) aged 0-<1 month, <100 bpm aged 1-35 months, <80 bpm aged 36-155 months, <70 bpm aged 156-179 months; Tachycardia defined as >150 beats per minute (bpm) aged 0-<1 month, >140 bpm aged 1-11 months, > 120 bpm aged 12-35 months, >110 bpm aged 36-155 months, >100 bpm aged 156-179 months

^6^ Circulation impaired=cold extremities and/or capillary refill time ≤2 sec. and/or tachycardia

^7^ Bradypnea defined as <46 breaths per minute (brpm) aged 0-<1 month, <34 brpm aged 1-11 months, <16 brpm aged 12-35 months, <20 brpm aged 36-71 months, <16 brpm aged 72-155 months, <12 brpm aged 156-179 months^f^; Tachypnea defined as <46 breaths per minute (brpm) aged 0-<1 month, <34 brpm aged 1-11 months, <16 brpm aged 12-35 months, <20 brpm aged 36-71 months, <16 brpm aged 72-155 months, <12 brpm aged 156-179 months

^8^ Diarrhea >7 days

^9^ Dehydration ≥dehydration grade 1 (3-5%)

^10^ Subjective interpretation of clinician

^11^ Leukocytosis=white blood cell count ≥10,000 /µl; Leukopenia=white blood cell count <4,000 /µl

^12^ Anemia: Hemoglobin <11 aged <7 years, Hemoglobin <12 aged 7-14 years

^13^ Number of children living in the family
